# The Role of Ovotransferrin in Egg-White Antimicrobial Activity: A Review

**DOI:** 10.3390/foods10040823

**Published:** 2021-04-10

**Authors:** Julie Legros, Sophie Jan, Sylvie Bonnassie, Michel Gautier, Thomas Croguennec, Stéphane Pezennec, Marie-Françoise Cochet, Françoise Nau, Simon C. Andrews, Florence Baron

**Affiliations:** 1 STLO, INRAE, Institut Agro, 35042 Rennes, France; julie.legros@agrocampus-ouest.fr (J.L.); sophie.jan@agrocampus-ouest.fr (S.J.); michel.gautier@agrocampus-ouest.fr (M.G.); thomas.croguennec@agrocampus-ouest.fr (T.C.); stephane.pezennec@inrae.fr (S.P.); marie-francoise.cochet@agrocampus-ouest.fr (M.-F.C.); francoise.nau@agrocampus-ouest.fr (F.N.); 2 School of Biological Sciences, Health and Life Sciences Building, University of Reading, Reading RG6 6AX, UK; s.c.andrews@reading.ac.uk; 3 UFR Sciences de la vie et de L’environnement, Université de Rennes 1, 35000 Rennes, France; sylvie.bonnassie@univ-rennes1.fr

**Keywords:** ovotransferrin, egg white, antimicrobial properties, iron chelation, membrane disturbing, *Salmonella* Enteritidis

## Abstract

Eggs are a whole food which affordably support human nutritional requirements worldwide. Eggs strongly resist bacterial infection due to an arsenal of defensive systems, many of which reside in the egg white. However, despite improved control of egg production and distribution, eggs remain a vehicle for foodborne transmission of *Salmonella enterica* serovar Enteritidis, which continues to represent a major public health challenge. It is generally accepted that iron deficiency, mediated by the iron-chelating properties of the egg-white protein ovotransferrin, has a key role in inhibiting infection of eggs by *Salmonella*. Ovotransferrin has an additional antibacterial activity beyond iron-chelation, which appears to depend on direct interaction with the bacterial cell surface, resulting in membrane perturbation. Current understanding of the antibacterial role of ovotransferrin is limited by a failure to fully consider its activity within the natural context of the egg white, where a series relevant environmental factors (such as alkalinity, high viscosity, ionic composition, and egg white protein interactions) may exert significant influence on ovotransferrin activity. This review provides an overview of what is known and what remains to be determined regarding the antimicrobial activity of ovotransferrin in egg white, and thus enhances understanding of egg safety through improved insight of this key antimicrobial component of eggs.

## 1. Introduction

Eggs are consumed all over the world, with an annual production of about 70 million tonnes [1]. The nutritional qualities of eggs (sources of proteins and lipids) as well as their low production cost make them an appealing whole food. Eggs can be consumed whole or as egg products (e.g., egg yolk, egg white, and whole egg in liquid, dried, or frozen forms). Thanks to their functional and organoleptic properties, eggs and egg products are utilised as ingredients in many food products (sauces, meat, seafood, dairy products, etc.) [2]. However, food poisoning resulting from bacterial contamination represents a major hindrance to the egg-food industry. *Salmonella* is implicated in 93% of reported foodborne outbreaks that are caused by the consumption of eggs or egg products. The main strain involved is *Salmonella enterica* serovar Enteritidis (66.7%) [3]. Such infection may arise from contamination of the eggshell after laying by microorganisms present in the laying environment, including those associated with hens’ faeces. Contamination by *Salmonella* Enteritidis can also occur during egg formation, in the oviduct or in the ovary, if the hen is infected. However, the egg possesses natural defences that protect against invasion of its internal compartments by bacteria. Understanding how bacteria can survive within egg white, and how egg white resists bacterial infection, are of clear importance to microbiologists, as well as to the agricultural and food industries, and are of great interest to the general public and government [4].

The first line of defence for the egg is the cuticle and shell, which act as physical barriers [5,6]. Over time and due to poor egg handling, the cuticle may rupture, allowing bacteria to penetrate the pores of the shell. Beneath the shell, the outer and inner membranes represent effective anti-bacterial “filters” that are composed of glycoprotein fibres, organized into a mesh, carrying traces of antibacterial molecules such as lysozyme [7]. Another key defensive element is the egg white, which contains an arsenal of antimicrobial molecules and displays properties that limit bacterial growth and migration into the egg yolk. The vitelline membrane contains specific proteins such as lysozyme and ovomucin, and represents the final obstacle for infection of the egg yolk [8]. Those bacteria that manage to reach the egg yolk are rewarded by ready access to a pool of nutrients and consequently enjoy rapid growth if the temperature is permissive (i.e., ~25 °C) [9].

The bacteria that contaminate the egg surface are mostly Gram-positive, but it is Gram-negative bacteria that are largely involved in the internal infection of eggs. This effect may be partly due to the high motility of Gram-negative bacteria and to their ability to resist egg white antimicrobial activities [10,11]. Indeed, Gram-negative bacteria, and particularly *S.* Enteritidis, have been described as being more resistant than other microorganisms to the egg’s natural defences [5,12,13].

When eggs are processed into egg products, the risk of contamination of the egg contents is increased, and so egg products are systematically pasteurized by heat treatment. Unfortunately, the heat sensitivity of egg white proteins restricts the pasteurisation process to a modest heat regime that is insufficient for effective eradication of all bacteria. Fortunately, egg white possesses various intrinsic antimicrobial activities and only a few Gram-negative bacteria (in particular, *S.* Enteritidis), can survive in egg white. The resistance of *S.* Enteritidis may explain the relatively high incidence of *Salmonella* in food poisoning outbreaks [5]. *Salmonella* survival or growth in egg white is very much influenced by incubation temperature ([4] for a review). At refrigerated temperatures, the growth of *Salmonella* in egg white appears entirely restricted. Between 20 and 30 °C, slight growth can be achieved in egg white, but for temperatures around 37–40 °C, a bacteriostatic or bactericidal effect is observed, depending on the precise temperature, incubation time, strain, and inoculum levels used. At 42 °C, the body temperature of the hen (i.e., as encountered in the oviduct during egg formation), egg white exerts a clear bactericidal effect ([4] for a review). 

Egg white is composed of 88% water, 10.6% proteins, 0.9% carbohydrates, and 0.5% minerals, but is heterogeneous in nature [2]. Several hundred proteins have been identified in egg white [14,15,16], and a number of these have confirmed antimicrobial properties ([4] for review). Some, such as lysozyme and defensins, cause damage to the bacterial envelope [17,18,19]. Others act by inhibiting bacterial proteases (ovostatin, cystatin, ovalbumin X) [20,21,22] or by limiting availability of key nutrients (e.g., avidin acts as a vitamin chelator, forming a complex with biotin [23]). Egg white also includes substantial quantities of ovotransferrin (13 g/L), a metal-chelating protein belonging to the transferrin family. 

It is generally accepted that iron deficiency, which results from the strong iron-binding activity of ovotransferrin, is the key process in the defence of egg white against microbial invasion [12,24,25,26]. Among the egg white proteins that display antimicrobial activity, ovotransferrin is the only one known to inhibit the growth of *S.* Enteritidis [25]. Further, there is evidence suggesting that ovotransferrin has an additional antibacterial activity, which is independent of its iron-restriction activity. This additional activity appears to require direct interaction with the bacterial-cell membrane and induces either destabilisation, permeabilisation, or damage of the membrane resulting in perturbed membrane function [27,28,29]. Interestingly, although it is well recognised that exposure of bacteria to whole egg white results in major perturbation of bacterial membranes [13,30,31,32,33,34], the specific contribution of ovotransferrin to this process (with respect to the overall activity exerted by egg white) has yet to be explored.

Other parameters also play a role in the passive immunity of egg white and can modulate the activity of egg white antimicrobial proteins such as ovotransferrin. Indeed, egg white presents specific conditions of pH, viscosity, ionic composition, and protein activities that could markedly influence the antibacterial functionality of ovotransferrin ([4] for a review). The high viscosity of egg white can limit bacterial mobility and accessibility to nutrients, including iron. Egg white pH increases from 7.8 to 9.3 over the 2–3 days post-laying period at room temperature [35]. This raised pH environment is generally recognised as being part of the antimicrobial activity of egg white [26,36], which could influence the activity of antimicrobial molecules as well as bacterial membrane status. The particular mineral composition of egg white (in addition to iron unavailability) and the presence of other proteins could also influence the antimicrobial effect of ovotransferrin. In addition to its antimicrobial activity, ovotransferrin plays other roles in protecting the development of the embryo by the regulation of iron absorption, and through its anti-viral and anti-inflammatory properties ([37] for review). This is why ovotransferrin and peptides resulting from its hydrolysis have been extensively reviewed and studied, in particularly to consider pharmaceutical or functional/nutraceutical food applications ([37] for review). 

This review focuses on the antimicrobial properties of ovotransferrin within egg white, rather than those properties often described in the literature where the conditions employed may not be representative of those found in egg white. The aim of this review is therefore to summarise the antimicrobial mechanisms of ovotransferrin that are already described and to highlight those that have yet to be demonstrated in egg white. The antimicrobial effects considered here will include those relevant to all bacteria, but with an emphasis on those observed for Gram-negative bacteria, particularly *S.* Enteritidis, since such bacteria are more resistant to egg white and more commonly infect eggs. Firstly, the structure of ovotransferrin will be briefly described with emphasis on the metal-binding sites that enable the key antibacterial property of the protein. The contribution of ovotransferrin to egg white antimicrobial activity will then be addressed, and the two distinct antibacterial mechanisms (chelation of iron and perturbation of bacterial membranes) of ovotransferrin will be considered. Finally, we explore the impact of specific environmental factors, as encountered by bacteria in the context of egg white, on the antibacterial mechanisms of ovotransferrin. Better comprehension of the mechanisms of ovotransferrin induced antibacterial effect in the egg white context is relevant to the eggs production sector and to food safety in view of the implication of the eggs and egg products in foodborne outbreaks.

## 2. Structure of Ovotransferrin

Ovotransferrin, also called conalbumin, is a monomeric glycoprotein belonging to the transferrin family that represents 13% (170 µM in egg white) of total egg white protein. Transferrins are extracellular glycoproteins involved in iron homeostasis, and they appear to have arisen from a common ancestorial gene through a gene duplication and fusion event that generated an encoded protein with two homologous lobes, each binding a single iron atom [38]. Four major types of iron-binding transferrin are known: Blood transferrin (serotransferrin); milk lactotransferrin (lactoferrin); membrane-associated melanotransferrin; and egg white ovotransferrin [39]. Although these four types of transferrin have distinct physiological roles, they all serve to control iron levels in biological fluids and thus possess conserved characteristics. Production of ovotransferrin in eggs is enabled as the gene is under the control of steroid hormones (oestrogen, progestins, glucocorticoids, and androgens [38]), which provides high-level ovotransferrin secretion into the egg white during its biosynthesis. 

**Figure 1:**
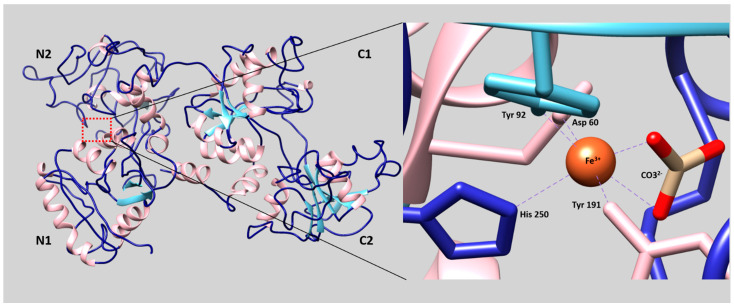
Representation of the tertiary structure of ovotransferrin. The subdomains of each lobe are indicated by N1, N2, C1, and C2. On the left, an enlargement of the binding site of the N lobe shows the binding of iron by its six ligands. The images were produced using the UCSF chimera package [45] from the tertiary structure of the ovotransferrin (PDB ID: 1AIV) [42].

From a structural point of view, hen ovotransferrin is composed of a single 686 amino acid residue polypeptide with a molecular mass of around 77.7 kDa and has an isoelectric point of 6 [40,41]. The two lobes of ovotransferrin (referred to as the N- and C-terminal lobes) each consist of two α/β domains (N1 and N2, and C1 and C2, respectively) of ~160 residues that are connected via two anti-parallel β-strands [42,43]. The amino acid sequences of the two lobes are similar, with a sequence identity of 37.4% [44]. A nine amino acid residue α-helix connects the two lobes (Figure 1).

The two iron-binding sites are located in the interdomain cleft in each of the corresponding lobes (Figure 1). Each lobe reversibly binds one Fe^3+^ cation along with one CO_3_^2−^ anion. Thus, two iron ions can bind to ovotransferrin in the presence of bicarbonate [43]. The interdomain clefts are in an open configuration under iron-free conditions, but adopt a closed conformation when they engage iron [46,47]. Binding of each iron atom is achieved via six ligands: Two are oxygen atoms from the bidentate carbonate ion and the four others are amino acid residues (Asp 60/395, Tyr 92/431, Tyr 191/524, and His 250/592, for the N-/C-lobes, respectively) [41,42,48,49]. The N- and C-lobes have distinct iron-affinity constants of 1.5 × 10^14^ and 1.5 × 10^18^ M^−1^, respectively [41]. This difference in iron affinity has been ascribed to lobe-specific amino acid residue interactions within the domains [42]. Iron-free ovotransferrin (apo-form) is more sensitive to physical, thermal, and chemical treatments than iron-bound ovotransferrin (holo-form) [50]. Ko and Ahn (2008) studied the effect of addition of iron (25–300% saturation) to ovotransferrin and found that 200% saturation prevented the denaturation of ovotransferrin with ethanol [51]. 

In addition to iron, ovotransferrin is able to bind other divalent cations such as chromium, copper, manganese, zinc, nickel, cobalt, and cadmium, but with lower affinities than for iron [52]. 

## 3. Role of Ovotransferrin in Iron Deficiency

### 3.1. The Requirement for Iron

Iron is essential for all forms of life, including bacteria, since it is involved in many cellular processes such as respiration, DNA synthesis, redox-stress resistance, and the tricarboxylic acid cycle (TCA) [53]. Concentrations varying from 0.1 to 10 µM are typically needed for optimal bacterial growth [53]. Iron is found in two major forms, either oxidized ferric iron, (Fe^3+^) or the reduced ferrous form (Fe^2+^). The ferric form is the most abundant under aerobic environmental conditions. However, ferric iron displays much lower solubility than the ferrous form (10^−18^ and 0.1 M at pH 7, respectively) [53]. On the other hand, the ferrous form is predominant and more stable in anaerobic environments, as well as under acid conditions. 

The addition of iron was shown, as early as 1944, to counteract bacterial growth inhibition in egg white. This effect was observed for several microorganisms, including *Escherichia coli*, *Shigella dysenteriae*, and *Staphylococcus aureus* [54]. In 1946, ovotransferrin was identified as the key factor limiting bacterial growth in egg white [55]. Assuming that (i) one mole of ovotransferrin is able to bind two moles of iron, and (ii) egg white contains around 3.6 to 18 µM of iron [35,56,57,58,59], it can be assumed that ovotransferrin is 1.07 to 5.4% iron-saturated suggesting that there would be virtually no free iron in egg white. Several studies have validated the iron-restriction role of ovotransferrin on bacterial growth. This effect was tested with different species, including *Pseudomonas fluorescens*, *Proteus vulgaris, Proteus melanovogenes*, and *Aerobacter cloacae* [60]. Garibaldi [60] showed that the bacterial population increased up to 8 log_10_ CFU/mL in a few days at 28 °C after the addition of iron at a concentration higher than the saturating concentration of ovotransferrin in egg white (addition of 20 mg/L of free Fe^2+^, leading to a 105% theoretical value of ovotransferrin saturation). Lock and Board (1992) showed that different *Salmonella* serotypes were able to remain viable in egg white at 20 °C and 30 °C for 42 days, and that the subsequent addition of 8 mg/L of ferric ammonium citrate (40% theoretical ovotransferrin saturation) induced growth by 4 log_10_ at both temperatures [12]. Baron et al. (1997) used egg white filtrate (obtained by egg white ultrafiltration at a molecular weight cut-off of 10 kDa) combined with individual egg white proteins to investigate which egg white protein contribute to the antimicrobial activity of egg white [25]. Only the addition of ovotransferrin resulted in a bacteriostatic impact on *S*. Enteritidis. Further, the authors showed that the addition of iron at 110% ovotransferrin saturation enhanced *Salmonella* growth by 4 log_10_ at 30 °C. Such studies demonstrate the major role of ovotransferrin in the anti-*Salmonella* effect of egg white through its iron deprivation action. Thus, ovotransferrin, like other transferrins, in addition to its host-iron transport and storage role, has a clear role in limiting the access of pathogenic bacteria to iron. However, bacteria have developed various mechanisms to sequester or scavenge iron from the host environment and from host-chelating proteins such as transferrins. 

### 3.2. Iron Metabolism in Bacteria

Intracellular iron concentration must be finely regulated, and a balance between iron uptake and storage must be maintained to ensure bacterial survival. When iron is restricted in the environment, bacteria often respond by secreting high-affinity iron-chelating molecules called siderophores [61], which have affinity constants for iron between 10^30^ and 10^52^ M^−1^ [53]. Over 500 siderophores are known and they are divided into several families according to the functional groups utilized in iron binding. The three major families are the catecholate, hydroxamate, and α-hydroxycarboxylate siderophores. The Enterobacteriaceae secrete enterobactin (also called enterochelin), which belongs to the catecholate family. Depending on the species or strain, Enterobacteriaceae also secrete salmochelin (glucosylated enterobactin), aerobactin (dihydroxamate), and yersiniabactin (a five-member heterocyclic siderophore) [53].

**Figure 2:**
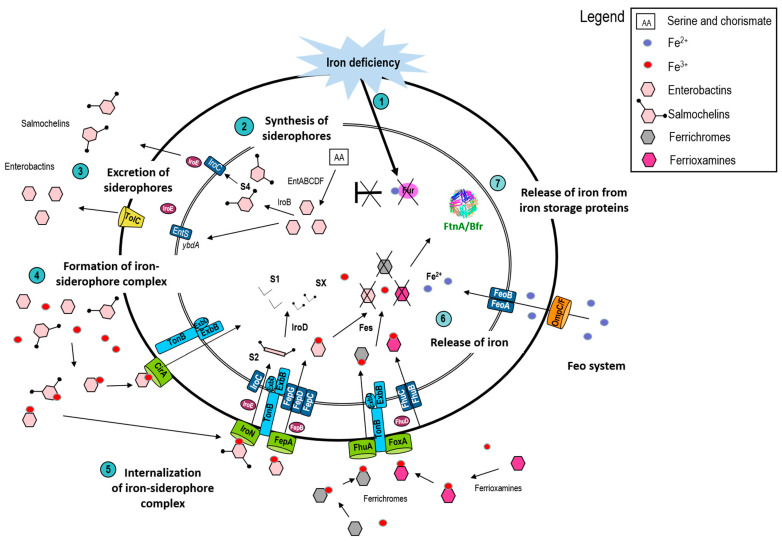
The different pathways of iron acquisition and storage in *S*. Enteritidis. (1) Iron deficiency induces derepression by the Fur regulator, leading to transcriptional upregulation of the genes under its control, in particular the genes encoding enzymes required for the production of siderophores. *Salmonella* is able to synthetize and/or use several siderophores: Enterobactin, salmochelin, ferrichrome, and ferrioxamine. (2) In the first step, enterobactin is synthetised using serine and chorismate as precursors. This step is catalysed by EntABCDF enzymes [62]. Then, enterobactin is transported into the environment by EntS and TolC or used as a precursor in the production of salmochelin S4 via the glycosyltransferase IroB [73]. (4) Once in the environment, the siderophores chelate ferric iron. (5) The siderophore-iron complexes (ferri-enteribactin and ferri-salmochelin, and the exogenous siderophores ferrichrome and ferrioxamine) are recognized by specific receptors present in the bacterial outer membrane (CirA and FepA for enterobactin, IroN for salmochelin, FhuA for ferrichromes, and FoxA for ferrioxamine) [74]. Then, the TonB-ExbBD, an energy-transducing complex, drives siderophore internalization into the periplasm. Once in the cytoplasm, salmochelin is linearised into the S2 form by IroE, an esterase [68]. The passage through the inner membrane is achieved by the FepBDGC transporter for both salmochelin and enterobactin [75,76]. On the other hand, ferrichromes and ferrioxamines use the FhuBCD transporter to pass through the inner membrane [77]. (6) Inside the cytoplasm, the iron-siderophore complexes are dissociated by esterases: Fes converts tricyclic enterobactin into monomeric units; IroD act on the linear, trimeric S2 form of salmochelin [68] to generate mono (S1) or dimeric (SX) products from which iron can be released more readily. Finally, iron can be utilized for metabolism or (7) stored by ferritin (FtnA) or bacterioferritin (Bfr) [78]. To acquire Fe^2+^, *S*. Enteritidis primarily uses the FeoABC system [71,77], which allows iron to be imported across the inner membrane from the periplasm; the OmpC and OmpF porins allow passive diffusion of ferrous iron across the OM [79].

The synthesis of siderophores takes place in the cytosol. Figure 2 shows the proteins involved in the iron-acquisition systems of *S*. Enteritidis. The enzymes required for enterobactin synthesis are encoded by the ent genes [62]. Enterobactin also acts as the precursor of salmochelin synthesis. The glucosylation of enterobactin, to form salmochelin, requires the product of the *iroB* gene [63]. It is important to emphasise that the glucosylation of enterobactin is a virulence strategy since it allows circumvention of the capture of enterobactin by siderocalin (also named lipocalin 2), a host innate immunity protein in human serum [64]. Siderocalin inhibits enterobactin-mediated bacterial growth during infection through its ferri-enterobactin sequestration activity [65]. Aerobactin synthesis is enabled by the *iucABCD* gene products, but this capacity is only found in some Enterobacteriacea [66]. Following synthesis, siderophores are secreted into the environment via specific exporters (Figure 2). In Gram-negative bacteria, the resulting ferri-siderophores complexes are transported across the outer-membrane (OM) by high-affinity and highly specific OM receptors (e.g., FepA for enterobactin). Subsequent translocation into the periplasm requires the energy-transducing TonB-ExbBD complex which utilizes the proton motive force (pmf) of the cytoplasmic membrane to drive ferri-siderophore transport [67]. Ferri-siderophores are transported from the periplasm through the cytoplasmic membrane via a periplasmic-binding protein-dependent ABC-binding cassette permease (e.g., FepBDGC for enterobactin). In the case of ferri-enterobactin, enterobactin esterase (Fes) facilitates iron release through degradation into dihydroxybenzoylserine units [68]. The reduced, ferrous form of iron can be acquired by *Salmonella* and other Enterobacteriaceae through the FeoABC (or SitABCD, which has preference for Mn^2+^) system, in particular under anaerobic conditions [66]. Ferrichrome and ferrioxamine are not synthesized by *Salmonella* but they can be recognized via specific receptors (Figure 2) and utilized for iron acquisition by *Salmonella* [69].

Iron uptake by Enterobacteriaceae is finely controlled by the ferric uptake regulator (Fur) protein, which acts as the global regulator of iron homeostasis according to cellular iron availability ([70] for a review). Fur represses iron-uptake systems, but also activates the expression of other genes [71] by either direct or indirect mechanisms, the latter involving repression of the small regulatory non-coding RNA, RyhB [72]. Genes repressed by Fur include those involved in iron transport and biosynthesis of siderophores; those induced include genes encoding iron storage, redox stress response, TCA cycle, and glycolysis functions. Furthermore, when bacteria are subject to iron restriction they can reduce their iron requirements by replacing iron-dependent proteins with non-iron alternatives and by down regulation of iron-demanding systems (“iron rationing”); they can also utilize intracellular iron stores in place of external iron ([70] for a review).

### 3.3. Capacity of Siderophores to Capture Iron from Ovotransferrin

By chelating iron, ovotransferrin leads to iron deficiency and to activation of high-affinity iron-acquisition systems. Studies on the impact of ovotransferrin-dependent iron restriction on the growth of *E. coli* and *S*. Enteritidis at 37 °C in Trypticase Soy Broth medium (TSB, pH 7) showed that ovotransferrin (5 g/L) induces expression of siderophore outer-membrane receptor proteins [80,81]. Further, ovotransferrin (1 g/L) severely impaired the minimal medium growth of *E. coli* mutants (Δ*fes*Δ*iroD* and Δ*fes*Δ*iroD*Δ*iroE*) lacking the esterases required for release of iron from enterobactin and salmochelin. However, the growth of single esterase mutants was similar to that of the wildtype [82]. These studies highlight the ability of ovotransferrin to trigger induction of ferri-siderophore acquisition systems and demonstrate that the function of only one such system is required to enable bacteria to counter the growth restriction imposed by ovotransferrin in laboratory media.

Recent studies, using a range of approaches, show that iron-acquisition genes are amongst the most highly induced when *S.* Enteritidis is exposed to egg white. Baron et al. (2017) used microarray analysis to study the global transcriptomic response of *S*. Enteritidis to egg white model medium (EMM) (10% of egg white in egg white filtrate) [83]. RNA-Seq analysis was used by Huang et al. (2019) to study the global gene-expression response of *S*. Enteritidis incubated in distilled water with 80% egg white [33]. Qin et al. (2019) used iTRAQ-based proteomics to examine protein secretion by *S*. Enteritidis in LB medium with 0 to 80% egg white [84]. All three studies found high-level induction of genes (or proteins synthesis) related to enterobactin (*entABCEFH*) and salmochelin (*iroBCDEN*) biosynthesis, and to the internalization of iron-siderophores complexes (e.g., *fepABCDG*, *cirA*, *fhuABCDE*, *tonB*, and *exbBD*). These studies clearly show that siderophore-related genes/proteins are strongly induced by *S.* Enteritidis upon exposure to egg white. However, such studies do not indicate whether siderophore production and utilisation provide any advantage for the survival and/or growth of *S.* Enteritidis in egg white.

An insightful study investigated the impact of combined deletion of both the ferrous and ferric uptake systems on the survival of *S*. Enteritidis in egg white at 37 °C [26]. Thus a double Δ*entF*/Δ*feoAB* mutant (lacking any effective high-affinity iron transport activity), was able to survive similarly to the wildtype in egg white at 37 °C, but after 70 h incubation, the mutant displayed a 2 log_10_ reduction in viable cells with respect to the wildtype. Although the addition of iron to the egg white (at 110% ovotransferrin saturation) enhanced growth of the mutant (and wildtype), the single Δ*entF* and double Δ*entF*/Δ*feoAB* mutants exhibited an extended growth lag, as well as reduced overall growth in comparison to the wildtype (3 to 5 log_10_ reduction, respectively). These results suggest that the production of siderophores by *S.* Enteritidis in egg white provides an advantage, especially when ovotransferrin is iron saturated such that iron availability is increased. Importantly, the siderophore-producing wild-type was unable to grow in egg white in the absence of iron supplementation. This suggests that *S*. Enteritidis is unable to access iron bound to ovotransferrin in egg white, despite its siderophore-production capacity. Indeed, egg white carries sufficient iron (3.6 to 18 µM [2,35,56,57,58]) to support bacterial growth, but this iron is likely to be almost entirely chelated by ovotransferrin since it is present in considerable molar excess. Thus, bacterial growth within egg white requires a suitable mechanism to acquire iron from ovotransferrin.

Garibaldi (1970) showed that the direct provision of siderophore compounds can overcome the bacteriostatic effect of egg white to allow rapid growth for *Salmonella* Typhimurium and *Pseudomonas ovalis* [24]. Neither species could grow in egg white, even when iron was added at up to 4 µg/mL, a concentration that is below that required to saturate ovotransferrin (~20% iron saturation was achieved). However, rapid growth was observed when siderophore-containing extracts were added to egg white, even without co-supplementation with iron [24]. These results therefore suggest that the levels of siderophore produced by bacteria in egg white may be insufficient to enable effective competition with ovotransferrin for iron such that the siderophore-production capacity of bacteria may provide no growth benefit.

Three lipocalins have been identified in egg white [14,15,85,86,87]. One of them, the “extra-fatty acid binding protein” (Ex-FABP), has been demonstrated to have a siderophore-sequestering activity [88]. However, although Ex-FABP can sequester enterobactin, it displays no activity against the di-glucosylated derivative of enterobactin, salmochelin [88,89]. The addition of Ex-FABP at the concentration found in egg white (5 μM) caused defective growth (1.5–2-fold reduction of OD 600 nm) of an *S*. Enteritidis *iro* (Ent^+^, Sal^−^) mutant using enterobactin as sole siderophore in an iron-restricted medium (LB with 2-2′ dipyridyl; DIP, a strong iron chelator) compared to the wildtype [88]. Thus, the sequestration of enterobactin by Ex-FABP reduced the bacterial iron-scavenging capacity. An *entB* mutant (unable to produce any siderophore) propagated under the same conditions exhibited a major growth defect (4-fold and 2-fold OD reduction cf. the wildtype and *iro* mutant, respectively). Thus, salmochelin production allows *S.* Enteritidis to overcome the growth inhibition that results from enterobactin sequestration by Ex-FABP under iron restriction. These findings suggest that the presence of Ex-FABP in egg white at ~5 µM may limit the ability of siderophores, such as enterobactin, to support growth in egg white.

To summarise, studies on the antimicrobial properties of egg white clearly demonstrate that egg white imposes iron restriction on bacteria due to the presence of ovotransferrin; such an effect is also seen for *S.* Enteritidis despite its considerable capacity for egg white resistance. In cases where bacteria manage to persist in egg white, subsequent addition of iron enables rapid growth. The iron naturally present in egg white is likely to be almost entirely bound to ovotransferrin. Upon exposure to egg white, *S.* Enteritidis up-regulates genes encoding ferrous and ferric iron acquisition systems, including the genes specifying siderophore production, secretion, uptake, and utilization. While it is generally accepted that production of siderophores is important for survival in egg white, this production is not sufficient to support growth without addition of iron; this observation raises a number of questions. Are siderophores produced in egg white, and does such production enable acquisition of iron from ovotransferrin? If siderophores are produced in egg white, why do they not allow growth in egg white; is this because they are produced in insufficient quantity? Furthermore, although saturation of ovotransferrin with iron relieves bacterial growth inhibition, it is unclear what minimal degree of saturation is required for relief of ovotransferrin-mediated growth inhibition in egg white and what impact the availability of carbonate (and other synergist anions required for iron-binding by ovotransferrin) has upon this. It is also unclear whether the physicochemical parameters of egg white (such as pH or temperature) contribute to the production of siderophores and/or on their ability to wrest iron from ovotransferrin. 

## 4. Role of Ovotransferrin in Bacterial Membrane Perturbation

### 4.1. Evidence for a Direct Interaction of Ovotransferrin with the Bacterial Membrane

Several antimicrobial mechanisms other than, or in addition to, iron restriction have been assigned to ovotransferrin. Valenti et al. (1985) showed that iron saturation of ovotransferrin had no impact on its antimicrobial activity against *Candida albicans* and suggested a complex mechanism involving a direct interaction between ovotransferrin and *Candida* cells. This assumption was supported by the microscopic observation that ovotransferrin induces cell aggregation [90]. The need for direct interaction in order to elicit this effect was confirmed when separation of ovotransferrin from *C. albicans* cells using a dialysis membrane prevented the antimicrobial activity [28]. However, the manner in which ovotransferrin directly interacts with *C. albicans* cells remains unknown. In addition, transferrin and lactoferrin have been shown to mediate damage to the outer membrane of Gram-negative bacteria [27]. The lipopolysaccharides (LPS) of the outer membrane of Gram-negative bacteria are composed of three parts: Lipid A; the core; and the O-antigen. The negative charges of LPS, resulting from the presence of numerous phosphate groups in the lipid A and core oligosaccharide regions, are bridged by divalent cations (Ca^2+^ and Mg^2+^) that are known to be crucial for the integrity of the outer membrane. Indeed, chelation of divalent cations is known to permeabilize the outer membrane of Gram-negative bacteria, making it more permeable to antimicrobial compounds [91,92]. According to Elisson et al. (1988), human transferrin and lactoferrin can chelate the divalent ions present on the surface of the outer membrane of *E. coli* [27]. The similarities between the properties of ovotransferrin, and human transferrin and lactoferrin suggest that ovotransferrin may act similarly in sequestering divalent ions from the outer membrane, leading to membrane destabilization. Direct binding of ovotransferrin to the bacterial outer surface is suggested by treatment of *Bordetella pertussis* under iron-restriction conditions which resulted in co-association of ovotransferrin with the outer-membrane protein fraction [93], indicating direct binding of ovotransferrin to an outer-membrane protein.

Further evidence for interaction of ovotransferrin with the bacterial membrane was provided by Aguilera et al. (2003) who showed that ovotransferrin can permeabilize the inner membrane of *E. coli* (for both whole cells and derived liposomes) resulting in selective leakage of K^+^ ions (but not Na^+^ or H^+^). The potassium permeabilization resulted in abolition of Δψ but not ΔpH, and a consequential dissipation of the proton motive force (pmf), which decreased from −198 to −56 mV [29]. The pmf is derived from the sum of the transmembrane electric potential (ΔΨ) and proton gradient (ΔpH), and drives the production of energy by the F_0_F_1_-ATPase through phosphorylation of ADP to ATP [94]. Thus, loss of pmf, as caused by membrane destabilization, impairs energy generation. Such disruption also allows ions to freely diffuse across the membrane along concentration gradients. An intact cytoplasmic membrane supports cytosolic homeostasis, efficient energy production, and pmf-dependent transport and motility functions, whereas dysfunction of the cytoplasmic membrane can lead to cell death.

### 4.2. Bacterial Membrane Perturbation in the Natural Context of Egg White 

Exposure of *E. coli* and *S.* Enteritidis to egg white has also been shown to elicit membrane damage [33,93]. Atomic force microscopy [95] and transmission electronic microscopy [33] demonstrated that egg white induces disruption of the cell envelop and leakage of intracellular contents. However, the egg white components specifically responsible for this membrane perturbation are not yet defined. The observed membrane damage is well reflected by the induction of a set of cell-envelop stress genes in response to exposure of *S*. Enteritidis to egg white and by the egg white sensitivity of mutants with defects in cell envelope systems. Approaches used to demonstrate such effects have included in vivo expression technology (IVET) [30], microarray- and RNAseq-based transcriptomic analysis [83], quantitative proteomics [84], directed mutagenesis [31,33,96], random mutagenesis [13], and microarray-based transposon-library screening [32].

Many of the *S.* Enteritidis genes/proteins highlighted by these studies have roles in periplasm homeostasis and degradation of abnormal proteins [33,83], membrane permeability and replacing the general-diffusion porins with porins of smaller pore-size [13,83], maintenance of cell envelope integrity [33,83,84,96], LPS biosynthesis [13,31,32], cell-wall integrity and biosynthesis [30], remodelling of the peptidoglycan [83,96], and removal of antimicrobial compounds through multidrug-efflux [33,83]. The majority of these genes are under the control of the CpxAR regulator that responds to a wide variety of envelope perturbations, including those induced by exposure to antimicrobial molecules or peptides ([97] for a review). Deletion of CpxAR-encoding genes has demonstrated that CpxAR is a key regulator for *S.* Enteritidis survival under the natural alkaline conditions of egg white at 37 °C [33]. The observed induction of the CpxAR-regulated genes in egg white is consistent with an attempt by *S.* Enteritidis to combat the antimicrobial egg white components that mediate envelope damage. There are several egg white components that cause damage to the bacterial envelope, and these could therefore contribute to induction of CpxAR-regulated genes by egg white. Such components include: Avian β-defensin 11 (AvBD11) [18]; gallin [19]; lysozyme; and ovotransferrin. In addition, there are likely to be other egg white proteins or peptides with unknown function that also act on the bacterial envelope to induce the CpxAR regulon [4]. Important new insight into the components of egg white responsible for induction of the CpxAR controlled genes in *S*. Enteritidis was recently obtained by utilisation of egg white ultrafiltrates (3 and 9 kDa cut-off membranes) [96] whereby similar CpxAR responses were achieved with egg white and the egg white filtrates. Thus, since the filtrates lacked ovotransferrin yet still induced the CpxAR regulon, it is clear that neither ovotransferrin (nor any other protein above 3 kDa) are strictly required for the observed CpxAR induction. 

In contrast to the induction of CpxAR regulon by egg white, the *psp* genes of *S.* Enteritidis were only induced by egg white when proteins of >10 kDa were present [34]. The *psp* genes form a six-cistron operon that is induced by changes in membrane status and stresses that lead to the dissipation of the pmf [98]. The suggested purpose of the *psp* genes is to restore the pmf and the biological functions of the membrane [99]. Upon egg white exposure, the *psp* genes where shown to be subject to rapid (≤7 min) up-regulation followed by an expression decrease from 25–45 min [83]. In apparent contradiction to this finding, Huang et al. (2019) failed to observe any induction of the *psp* genes by egg white [96]; this discrepancy likely arises from the longer incubation times (6 and 24 h) employed.

To further explore any pmf disruption effect caused by egg white proteins, Baron et al. (2020) tested the membrane depolarization of *S.* Enteritidis [34] using the diSC3(5) fluorescent dye method [34,100]. This study showed a significant increase in the fluorescence of *S.* Enteritidis upon incubation in egg white, but not in egg white filtrate where egg white proteins of >10 kDa were absent [34]. The authors concluded that the proteins (>10 kDa) present in egg white are responsible for the observed depolarization of the bacterial membrane [34]. Although the identity of the proteins involved has not yet been determined, it was suggested that ovotransferrin may play a part since previous studies have shown a disruptive effect of ovotransferrin on bacterial membranes [27,29] and the electrochemical potential of the cytoplasmic membrane of *Bacillus cereus* [101].

To conclude, it is likely that ovotransferrin, like other transferrin-family members, is able to chelate divalent ions present on the surface of the outer membrane of Gram-negative bacteria and provoke membrane perturbation, probably by direct contact. The bacterial membrane perturbation effects induced by ovotransferrin include membrane permeabilization to potassium and dissipation of the pmf. In addition, several studies have shown that egg white induces the expression of *S.* Enteritidis genes (and/or the production of proteins) involved in the envelope-damage response, maintenance of membrane integrity, and pmf restoration. The mechanism(s) by which ovotransferrin induces perturbation of bacterial envelopes, under the specific conditions found in egg white, remain(s) to be clarified. Understanding these mechanisms, and the contribution that the specific conditions of egg white play in ovotransferrin-induced bacterial membrane perturbations, would be expected to greatly support research aimed at optimizing the control of egg contamination.

## 5. Impact of Egg White Conditions on Ovotransferrin 

### 5.1. Impact of Egg White pH 

In egg white, the pH increases from 7.8 to 9.3 a few days after laying [35]. This is caused by the loss of CO_2_ through the pores of the eggshell [102]. The maintenance of intracellular pH around its optimum value (7.4 to 7.8 [103,104,105]) is essential for many biological functions, particularly for bacterial enzymatic activities and the status of membranes. Excessive differences in pH between the environment and the bacterial cytoplasm can lead to energetically unfavourable conditions for growth [106]. The impact of egg-white pH on bacterial behaviour has been much reported. At 39.5 °C, Tranter and Board (1984) showed a bactericidal effect on *E. coli* incubated at pH 9.3, while bacteriostasis was observed at pH 7.8 [36]. At 37 °C, bacteriostasis was reported by Kang et al. (2006) for *S*. Enteritidis incubated at pH 9.0, although growth was seen at pH 8.0 [26]. At 20 °C, a one-log_10_ reduction of *S*. Enteritidis growth was observed at pH 9.3 compared to 8.2 [107]. Alabdeh et al. (2011) showed that alkalinization (increase in pH from 7.8 to 9.3) potentiates the antibacterial effect of egg white on *E. coli* and *S.* Enteritidis at temperatures that are growth permissive (between 20–37 °C) or bactericidal (above 40 °C) [108]. However, at 39.5 °C the addition of 20 µg/mL iron to egg white (corresponding to 105% ovotransferrin saturation) overcame the bactericidal effect at pH 9.0 and the bacteriostatic effect at pH 7.8 for *E. coli* [36], with iron supporting better growth at pH 7.8 than at pH 9. 

**Figure 3:**
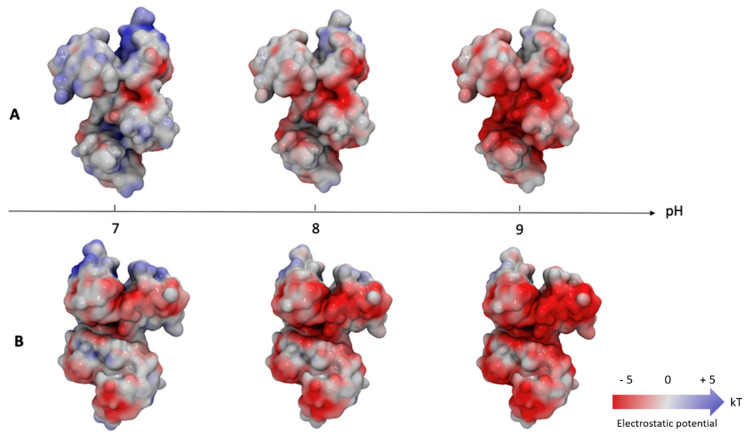
Prediction of electrostatic charge distribution at the surface of ovotransferrin. Each representation (**A**,**B**) corresponds to a rotation of the molecule by 180° around the vertical axis. Charges were computed at each pH using the PDB2PQR tool [110] and the electrostatic potential of the protein surface was estimated with the APBS tool [111], using a specific server (https://server.poissonboltzmann.org/ (accessed on 15 January 2021)). The molecule was visualised using the VMD software [112]. The surface of each molecule is coloured according to the electrostatic potential, from −5 kT (red) to + 5 kT (blue), via 0 kT (white), where k is the Boltzmann constant and T the absolute temperature.

The impact of pH (from 6.5 to 9) on iron-binding by ovotransferrin has been studied by Okamoto et al. (2004). They showed that lobe preference for initial iron binding is highly dependent on pH [109]. However, it remains unknown whether the pH of egg white (7.8 or 9.3) influences the overall metal chelating activity of ovotransferrin. Another pH impact of relevance is influence on the interaction between egg white proteins and the bacterial outer membranes. Figure 3 shows a structural model of apo-ovotransferrin (PDB ID: 1AIV) with its molecular surface coloured by the electrostatic potential calculated at pH 7.0, 8.0, or 9.0. This model indicates that the few positively charged areas present on the surface of ovotransferrin at pH 7.0 are almost completely lost at pH 9.0. Therefore, ovotransferrin appears as negatively charged at alkaline pH, whatever the surface area considered.

The impact of the pH-related change in surface charge on ovotransferrin function (e.g., interaction with bacterial surfaces and or metals ions) remains largely unexplored. Moreover, it was shown that ovotransferrin provokes a stronger depolarization of the cytoplasmic membrane of the Gam-positive *B. cereus* at pH 9.3 than at neutral pH [101]. The precise influence of egg white pH on the membrane perturbation activity of ovotransferrin requires further investigation (especially for *S.* Enteritidis).

### 5.2. Impact of the Ionic Composition of Egg White

Studies investigating the antimicrobial activity of ovotransferrin have been performed mainly in minimal or rich model media under a range of iron regimes. However, Baron et al. (1997) investigated the effect of ovotransferrin under the ionic environment of egg white by utilizing egg white filtrate (free of >10 kDa proteins) and found that ovotransferrin exerts a major antibacterial impact on *S.* Enteritidis [25]. However, it should be pointed out that the ion concentration of egg white (Table 1) is variable and the influence of metallic ions other than iron has not been explored. Indeed, ovotransferrin is able to bind iron, chromium, copper, manganese, zinc, and aluminium (listed in respective order of decreasing affinity) [111,112,113,114,115]. Valenti et al. (1987) found that 100% saturation of ovotransferrin with zinc increases its bacteriostatic activity in BHI medium at 37 °C, but that saturation of ovotransferrin with iron reverses the antimicrobial effect of the Zn-ovotransferrin complex [116]. These authors showed that iron, copper, and zinc ions are in competition for the same binding site in ovotransferrin, but they failed to explain why the bacteriostatic activity of ovotransferrin is enhanced by complexation with zinc [116]. The role for egg white metals (Table 1) in the antimicrobial activity of ovotransferrin has not yet been fully studied. The investigation of such effects is complicated by the need to consider changes in egg white metal content during the course of egg storage since fluxes of minerals are observed due to metal migration from the egg yolk through the vitelline membrane [117]. This could have an impact on egg white metal content and, consequently, on the antimicrobial activity of ovotransferrin. In addition, the ion content of eggs is influenced by diet such that variations of 28% are observed for manganese and zinc, 40% for copper, and 12% for iron [118].

According to the concentration ranges detected in egg white (Table 1), ovotransferrin appears to be only 1.07 to 5.4% saturated for iron, 0.48 to 5.4% for zinc, 0.87 to 1.74% for copper, and 0.38 to 0.60% for manganese; this gives a maximum total metal saturation of ~13%, which leaves a considerable residual chelation capacity.

Bicarbonate is present in egg white at around 55 mM and originates from the mother hen’s blood, as is the case for other ions that enrich egg white during hydration in the hen’s uterus [119,120]. Valenti et al. (1981) showed that the addition of 50 mM bicarbonate increases the antimicrobial activity of a 10 g/L ovotransferrin solution towards Staphylococci and *E. coli* incubated in BHI medium at 37 °C [121], presumably due to the dependence of metal-complex formation on the co-binding of anions such as bicarbonate [49]. Similar results were obtained with *E. coli* O157:H7 and *Listeria monocytogenes* incubated with 100 mM bicarbonate in BHI at 37 °C [122]. The combination of high bicarbonate and relatively low iron with ovotransferrin in egg white would be expected to greatly support the metal-chelation activity of ovotransferrin, ensuring very low access to iron for bacterial invaders. 

The mineral composition of egg white may support the integrity of the outer membrane due to the presence of divalent cations such as Ca^2+^ and Mg^2+^ that are bound to the LPS and contribute to resistance against antimicrobial agent. It remains unclear whether ovotransferrin within egg white has the capacity to remove these cations from bacterial outer membranes and if any such activity results in perturbation of the outer membrane. In conclusion, the mineral composition of egg white may have an impact on the antimicrobial activity of ovotransferrin. This possibility requires further exploration, especially in view of the potential for optimizing the antimicrobial activity of ovotransferrin through influencing the ionic composition of egg white by suitably adjusting hen feeding and/or egg-storage practices.

### 5.3. Impact of Viscosity and the Heterogeneous Structure of Egg White

Egg white has a viscosity of 5 mPa.s^−1^ at 20 °C and a shear rate of 400 s^−1^, which corresponds to a highly viscous medium [123]. However, egg white viscosity is not homogeneous with respect to its three distinct layers: There are two layers of low-viscosity (thin) egg white, one located near the egg yolk and the other close to the eggshell; and a layer of high-viscosity (thick) egg white sandwiched in between the “thin” layers. The thick egg white can be 40 times more viscous than the thin egg white [123] due to the presence of ovomucin, which forms filamentous super-aggregates [124,125]. The viscosity of egg white decreases during egg storage due to biochemical changes (not yet completely understood) involving ovomucin [124,126,127,128,129,130,131] which might impact infection potential. It is likely that the viscosity of egg white and the presence of heterogeneous rheological fractions (thin and thick layers) hinder bacterial infection by limiting motility resulting in reduced access to nutrients and dissemination throughout the egg. Moreover, several authors have suggested that iron-ovotransferrin complexes are probably not distributed uniformly within egg white [25,132]. It can be assumed that egg white viscosity and its heterogeneous structure will have an impact on bacterial contamination, and on the interaction between bacteria and ovotransferrin.

### 5.4. Impact of the Presence of Others Egg White Proteins

Synergistic or antagonistic effects can occur between ovotransferrin and other egg white proteins. Cooperation between lysozyme and lactoferrin has already been established for anti-microbial effects on Gram-negative bacteria [133,134,135]. Lysozyme hydrolyses the β(1–4) linkage between the N-acetylglucosamine and the N-acetylmuramic acid residues of peptidoglycan in both Gram-negative and Gram-positive bacteria. This hydrolysis activity results in lysis of Gram-positive bacteria under conditions where the internal osmotic pressures exceed those externally. However, the outer membrane surface of Gram-negative bacteria protects bacteria from lysozyme activity. The lysozyme resistance of Gram-negative bacteria can be impaired by the addition of molecules that chelate the divalent ions of the LPS layer, allowing peptidoglycan lysis and membrane damage [136,137,138]. A synergistic effect of lactoferrin and lysozyme has been observed in 1% of peptone medium [133,134]. According to Ko et al. (2008), the addition of 2.5 to 3 g/L lysozyme to a 20 g/L ovotransferrin solution exerts a synergistic bacteriostatic effect on *L. monocytogenes* (a Gram-positive bacterium) in BHI (pH 7) medium at 37 °C, but only in the presence of 100 mM bicarbonate [139]. In contrast, at 35 °C and in the same medium, no significant difference in bacterial growth was observed when *E. coli* (Gram negative) was incubated with ovotransferrin (20 g/L with 100 mM bicarbonate) with or without 1 g/L lysozyme [122]. However, these studies did not consider the synergistic effects of lysozyme and ovotransferrin under conditions that reflect those of egg white (i.e., at 13 and 3.5 g/L for ovotransferrin and lysozyme, respectively, and at alkaline pH and with relevant mineral levels).

Other work has shown that genes encoding proteins involved in the inhibition of lysozyme activity (lysozyme inhibitor: *ydhA* and *SEN1802*) are induced when *S.* Enteritidis is incubated with egg white proteins (45 min at 45 °C) [34]. It was suggested that exposure of *S.* Enteritidis to egg white proteins at 45 °C caused permeabilization of the outer membrane (through chelation of the divalent cations of the LPS layer by ovotransferrin) which enabled lysozyme to gain access to the peptidoglycan, stimulating the induction of the expression of the lysozyme inhibitor gene in response [34].

The possible synergy between ovotransferrin and lysozyme (or other egg white components) in mediating antibacterial activity remains to be clarified under the natural conditions of egg white. Such insight would enhance understanding of the antimicrobial activity of egg white and egg white preservation processes. Understanding the complex and effective synergy of egg-white proteins represents a strong basis to consider practical applications (such as hen selection and egg storage) for increasing antimicrobial egg white defence and enhancing egg safety.

### 5.5. Impact of Ovotransferrin-Derived Peptides 

In addition to the antimicrobial role of native ovotransferrin, antimicrobial peptides derived from ovotransferrin have also been identified. The acid hydrolysis of ovotransferrin yielded a cationic peptidic fragment (OTAP.92) which exhibited a wider antimicrobial spectrum than native ovotransferrin. The OTAP.92 peptide was found to kill bacteria by disrupting the function of the cytoplasmic membrane [140]. More recently, another peptide, designated OVTp12, was isolated from ovotransferrin (by pepsin hydrolysis) and found to increase the membrane permeability of bacteria [141]. However, although these ovotransferrin fragments display clear antibacterial activity, their relevance to egg white immunity is uncertain since they are unlikely to be present given the absence of the corresponding hydrolysis processes.

Nevertheless, protein degradation may occur during egg storage. An SDS-PAGE analysis highlighted the presence of proteolytic fragments after five-day storage of eggs at 37 °C [142], and peptides derived from the proteolytic degradation of ovotransferrin were identified by mass spectrometry [142]. Liu et al. (2018) showed that the overall protein content of egg white gradually decreases during egg storage at room temperature, with a corresponding increase in peptide content [143]. In the <3 kDa egg white fraction, a significant increase in peptide content was observed after 21 days of storage, and after 56 days, the peptide level was increased by 17-fold with respect to those of fresh eggs. Six ovotransferrin-derived peptides were subsequently identified in the <3 kDa egg white fraction from day 56. The authors suggested that storage at room temperature impacts ovotransferrin in a fashion that leads to its consequential degradation to generate the observed peptides [143]. However, it remains unclear whether these ovotransferrin-derived peptides have antibacterial activity under egg white conditions and what mechanism drives their formation.

### 5.6. Impact of Egg Processing and Technological Factors

During egg production, storage and processing, and egg-product manufacture, any operation that introduces iron (or other metals) or impairs ovotransferrin activity can potentially modulate the bacteriostatic activity of egg white. It is generally recognized that egg quality is influenced by egg production processes (housing systems, breeding practice, hen age, and hen nutrition), but the levels of the major egg components, including egg white proteins (and ovotransferrin), are relatively stable [117]. However, the mineral content of eggs can be altered by the hen diet [117]. Egg contents are particularly impacted by egg-storage factors such as temperature and duration. Shell eggs are generally consumed within 28 days after laying. During this period, eggs are stored at various temperatures depending on the site of storage (laying farm, conditioning centre, food store, consumers’ home) and might be subject to different temperatures generally ranging between 20 °C (ambient temperature) and 4 °C (refrigeration temperature). During egg aging, gas exchange (loss of CO_2_) with the external environment leads to egg-white alkalinisation. In addition, the thick-layer content and viscosity of the egg white decrease, and the degradation of the vitelline membrane allows mineral transfer between the albumen and yolk [117]. 

When eggs are incorporated into commercial foodstuffs, manufacturers utilise ready-to-use egg products. Egg white is widely used as an ingredient in various food products due to its whipping (cakes, meringues, and confections) and textural properties (sausages, terrines). The transformation of shell eggs into safe liquid or spray-dried egg white with extended shelf life requires technological operations that could impact the antibacterial activity of ovotransferrin. The first step of egg-white product manufacturing involves breaking the eggshell and separation of egg white from egg yolk. The integrity of the vitelline membrane is important at this point to ensure that the yolk is retained within the vitelline membrane to thus avoid any contamination of egg white with egg yolk. Baron et al. (1999) showed that the presence of yolk in egg white enhances *Salmonella* growth due to increased iron availability and ovotransferrin saturation [144]. They observed that egg white collected (just after breaking) from several factories allows *Salmonella* growth at similar levels to that observed experimentally in egg white containing 0.7% yolk. These findings highlight the need to ensure that the egg breakage and yolk separation processes are carefully controlled (considering factors such as egg quality, egg age, processing parameters, and operator vigilance) to avoid yolk contamination. 

After egg breakage, the isolated liquid egg white is pasteurised to enhance food safety. However, the heat treatment employed must be mild (traditionally 57 °C for 2 to 6 min) because of the thermal fragility of egg white, and such mild treatment is insufficient for effective *Salmonella* elimination [6]. However, dried egg white (powder) is used in place of liquid egg white by many sectors of the food industry for practical reasons (e.g., room storage and microbiological stability due to low water activity) which include the possibility of applying a much stronger and more effective heat treatment (incubation at 67 °C or 75 °C for 15 days) to the dried-egg product [6]. Heat treatment of egg-white powder has the dual advantage of ensuring effective *Salmonella* elimination and improvement of the functional properties (whipping and gelling) of the dried egg white functional properties are important for various foodstuffs including cakes, meringues, and surimi [6]. The egg-white drying process involves pumping and atomization (aerosol generation) and is considered to cause only minor changes to the egg-white proteins [145]. Nevertheless, Baron et al. (1999) showed that this pasteurization process results in a dramatic loss of bacteriostatic activity for egg white when it is subsequently reconstituted such that a rapid growth of *Salmonella* is observed in the reconstituted egg white (comparable to that obtained in optimum growth medium) [144]. This effect likely arises from the heat lability of ovotransferrin, which was found to possess the lowest heat stability of all the major egg-white proteins, with a denaturation temperature of 60 °C at pH 9 (for apo-ovotransferrin) [146]. Indeed, Baron et al. (2003) subsequently demonstrated that the observed loss of bacteriostatic activity is attributable to the thermal denaturation of ovotransferrin during pasteurization resulting in a reduction in its iron chelation activity [147]. 

It is therefore clear that technological factors applied during egg-white product manufacture can modify ovotransferrin activity, especially when iron is introduced or thermal treatment is applied. This is also the case when egg white protein is incorporated into other foods as an ingredient and then exposed to technological processes within the subsequent food chain.

## 6. Conclusions

Among the egg white factors involved in limiting the invasion of the egg by bacterial contaminants, ovotransferrin represents a key antimicrobial component. Ovotransferrin is known to have a role in limiting bacterial growth by iron deprivation due to its strong iron-chelation activity. However, it also causes membrane perturbation, which provides a second mechanism for limiting bacterial growth. Although ovotransferrin is much studied, its activity under the conditions associated with egg white is poorly described. Therefore, there remains a need to clarify the role of ovotransferrin in egg white defence against bacteria particularly with respect to key egg white factors such as alkalinity, high viscosity, ionic composition, potential synergistic action with other egg white proteins, and the impact of storage and technological practices on ovotransferrin degradation and activity. Indeed, variation in packaging and storage temperatures between countries is yet another consideration of relevance for ovotransferrin antibacterial activity. A more complete understanding of ovotransferrin activity in egg white may suggest modifications of hen diet and egg-storage practice that could improve the antimicrobial activity of ovotransferrin in egg white and thus reduce the risk of microbial contamination and food poisoning.

## Figures and Tables

**Table 1:** Mineral composition of egg white.

	Min Concentration in Egg White (mM) *	Max Concentrationin Egg White (mM) *
Sodium	67.42	80.91
Sulphur	50.83	56.14
Potassium	35.81	44.25
Phosphorus	4.2	7.10
Magnesium	3.70	4.94
Calcium	1.25	2.99
Chlorine	0.11	0.13
Iron	0.0036	0.0179
Zinc	0.0015	0.0185
Copper	0.0029	0.0058
Manganese	0.0013	0.0020

* The values are obtained from Nys and Sauveur, 2004; Sauveur 1988, Stadelman and Cotterill, 1995; Nau et al., 2010 and Ciqual, 2020 [35,56,57,58,59]. Whereas chloride, sodium, and potassium are mainly free in solution, sulphur is a constituent element of egg white proteins. Calcium and magnesium are partly bound to proteins and are heterogeneously distributed between thick and thin egg white.
